# Knockdown of Gene Expression in Macrophages by microRNA Mimic-Containing Poly (Lactic-*co*-glycolic Acid) Microparticles

**DOI:** 10.3390/medicines5040133

**Published:** 2018-12-15

**Authors:** Paul J. McKiernan, Patrick Lynch, Joanne M. Ramsey, Sally Ann Cryan, Catherine M. Greene

**Affiliations:** 1 Department of Medicine, Royal College of Surgeons in Ireland, Beaumont Hospital, Dublin 9, Ireland; pmckiernan3@gmail.com (P.J.M.); patricklynch@rcsi.ie (P.L.); 2 Drug Delivery and Advanced Materials Team, School of Pharmacy, Royal College of Surgeons in Ireland, Dublin 2, Ireland; joanneramsey@rcsi.ie (J.M.R.); scryan@rcsi.ie (S.A.C.); 3 Centre for Research in Medical Devices (CURAM), RCSI, Dublin and National University of Ireland, Galway H91 HE94, Ireland; 4 Trinity Centre for Bioengineering, Trinity College Dublin, Dublin 2, Ireland; 5 Tissue Engineering Research Group, Royal College of Surgeons in Ireland, 123 St Stephens Green, Dublin 2, Ireland; 6 Lung Biology Group, Department of Clinical Microbiology, Royal College of Surgeons in Ireland, Beaumont Hospital, Dublin 9, Ireland

**Keywords:** microRNA, microparticle, secretory leucoprotease inhibitor, macrophage

## Abstract

**Background:** microRNA (miRNA) regulate target gene expression through translational repression and/or mRNA degradation and are involved in the regulation of inflammation. Macrophages are key inflammatory cells that are important in chronic inflammatory lung diseases such as cystic fibrosis (CF). Macrophage-expressed miRNA represent therapeutic drug targets, yet delivery of nucleic acids to macrophages has proved challenging. **Methods:** miRNAs were encapsulated in poly (lactic-*co*-glycolic acid) (PLGA)-based microparticles using double emulsion solvent evaporation and characterised for physicochemical features. Phorbol myristic acetate (PMA)-differentiated U937 macrophages were transfected with empty PLGA microparticles or those encapsulating a premiR-19b-3p or scrambled control miRNA mimic. miRNA internalisation and knockdown of a miR-19b-3p target gene, secretory leucoprotease inhibitor (SLPI), were determined by qRT-PCR. **Results:** Microparticle formulations were consistently found to be 2–3μm and all had a negative ζ potential (−5 mV to −14 mV). Encapsulation efficiency of premiR-19b-3p was 37.6 ± 13.4%. Levels of mature miR-19b-3p were higher in macrophages after delivery of premiR-19b-3p microparticles compared to empty or scrambled control miRNA-containing microparticles. Significant SLPI knockdown was achieved 72 hours post-delivery of premiR-19b-3p microparticles compared to controls. **Conclusions**: miRNA-encapsulating PLGA microparticles offer a new treatment paradigm for delivery to macrophages that could potentially be administered to CF lungs via inhalation.

## 1. Introduction

Cystic fibrosis (CF) is a clinically complex, autosomal recessive genetic disease that occurs due to mutation of a single gene, the cystic fibrosis conductance regulator gene (*CFTR*). The principal symptoms of CF are an elevated sweat chloride concentration, progressive pulmonary disease, exocrine pancreatic insufficiency and male infertility [1]. The major pulmonary pathology is characterised by inadequate homeostasis of the airway surface layer and impaired mucociliary clearance. This leads to increased mucus viscosity and chronic bacterial infection. A vicious cycle of infection ensues, followed by intense neutrophil-dominated inflammation, ineffective clearance of infection and bronchiectasis and ultimately respiratory failure due to irreversible airway destruction [2]. 

It is well established that the CF lung is dominated by a neutrophilic inflammation. Although neutrophils are required for antimicrobial defence, their accumulation over time and poorly controlled release of their toxic granule content can lead to parenchymal lung tissue damage [3,4]. The high numbers of neutrophils in the CF lung results in a high concentration of proteases. Elevated levels of active neutrophil elastase (NE), the major protease released by CF neutrophils, are evident in both paediatric and adult CF bronchoalveolar lavage fluid (BALF) compared to healthy controls [5,6,7].

Antiproteases are a class of molecules that exist to counter–balance the over-exuberant and sometimes harmful effects of proteases. Secretory leucoprotease inhibitor (SLPI), alpha-1 antitrypsin (A1AT), and elafin are the three major lung serine antiproteases [8,9]. The primary role of SLPI is as a serine protease inhibitor; it can protect tissue from degradation by NE, cathepsin G, elastase, trypsin, chymotrypsin, chymase and tryptase. SLPI has also been shown to exhibit anti-inflammatory/immune-modulatory functions [10,11,12,13,14,15]. Recombinant SLPI, when administered in an aerosolised form to CF patients, can suppress airway NE levels and reduce levels of the neutrophil chemokine interleukin-8 (IL-8) in BALF obtained from these patients [16,17]. Thus, SLPI holds therapeutic potential via local administration to the lung for the treatment of CF [18]. However, aerosolised SLPI has a short half-life (~12 hours) and is unevenly distributed when ventilated into the lung [19], therefore, modulation of SLPI expression via other methods, such as by microRNA modulation, may be of therapeutic benefit in CF. 

microRNAs (miRNAs) are 20–25 nucleotide duplex RNAs involved in the translational regulation of gene expression [20]. MiRBase version 21 (http://www.mirbase.org, v21 [21], Manchester, UK) contains at least 2000 human microRNA entries. Levels of miRNAs can vary greatly between cells and tissues, and aberrant levels of miRNAs are associated with many diseases in humans including lung inflammation and disease [22]. miRNAs that are downregulated in disease, or which would be predicted to have therapeutic benefit if present at higher levels, may be replaced transiently by using miRNA mimics, or more stably using a transgene approach to deliver DNA encoding primary, pre- or mature miRNAs [23] generally via plasmid DNA. miRNAs that are overexpressed or have a gain-of-function in diseased tissue may be therapeutically targeted using, for example, anti-miRNA oligonucleotides (anti-miRs) based on locked nucleic acids [24] or cholesterol-conjugated anti-miRs termed ‘antagomiRs’ [25].

The ability to deliver naked miRNAs in vivo is restricted, as these and other small RNAs are polyanionic and highly susceptible to destruction by serum nucleases [26]. Non-viral vectors such as liposomes, dendrimers and synthetic and natural polymers have been explored to varying success in the delivery of miRNA therapeutics [27]. Polymers are widely and safely used in pharmaceutical products and medical devices and have been used to deliver miRNAs to target cells. Polymeric nanoparticles are increasingly being explored for pulmonary delivery of nucleic acids to the epithelium due to their advantages, including relative ease of delivery to the lungs using nebulisation [28]. However, alternative approaches must be considered for delivery to the more difficult to transfect monocyte/macrophage lineage. Monocytes originate from precursors in the bone marrow, and circulate in the bloodstream, until they are attracted to infection or inflammatory signals in particular tissues, such as the lung, where they differentiate into macrophage or dendritic cell populations [29]. Monocytes and macrophages produce SLPI [30].

Prior studies using cationic lipid-based systems and liposomes have been evaluated for delivery of nucleic acids to monocytes and macrophages, yet they have shown poor transfection efficiency [31,32,33]. Electroporation has achieved moderate transfection efficiencies in cell lines [33] but is not a clinically viable option in vivo. Poly (lactic-*co*-glycolic acid) (PLGA) microparticles have been previously engineered by our group to specifically target macrophages [34,35]. Microparticles within the range of 2–3 µm have been explored for nucleic acid delivery to macrophages by harnessing the phagocytic capacity of these cells as a mechanism for uptake. Microparticles have been used extensively in the delivery of DNA and small interfering (si)RNA to various lymphoid and myeloid cells via a number of routes, including intranasal, inhalation and intraperitoneal administration [36,37,38]. PLGA-based microparticles have also been widely explored for delivery of small molecules and protein-based therapeutics to the lung [39]. These microparticles allow for controlled release of drug cargo over long periods, thereby decreasing the frequency of repeated administration. In vivo PLGA undergoes degradation via hydrolysis of its backbone ester linkages into lactic acid and glycolic acid, which are subsequently removed via the citric acid cycle [40]. PLGA carriers loaded with nucleic acids may achieve sustained cytoplasmic delivery via rapid escape from endolysosomes once internalised [41,42]. However, poor encapsulation and transfection efficiencies have been observed when using unmodified PLGA microparticles [43]. These efficiencies can be improved through the addition of cationic excipients, such as the lipid 1,2-dioleoyl-3-trimethylammonium-propane (DOTAP), into the manufacturing process, and these can improve the miscibility between the drug and polymer [35,40]. Apart from the recent seminal work of Moore et al. [44], little has been done using PLGA in the delivery of miRNAs to macrophages. Here, the preparation, characterisation and delivery of miRNA-based PLGA microparticles is described to enable cell-type specific targeting within the lungs. Particles in the micron size range are used in this study with the aim of targeted delivery to macrophages. The efficiency of internalisation and target gene knockdown is examined in macrophages through the use of miR-19b-3p mimic-encapsulating PLGA microparticles. 

## 2. Materials and Methods 

### 2.1. Encapsulation of miRNA in PLGA-Based Microparticles

Microparticles containing the pre-miR-19b-3p mimic (Applied Biosystems, FosterCity, CA, USA, catalogue ID PM10629), scrambled non-targeting pre-miR mimic (Applied Biosystems, catalogue ID 4464058), or fluorescent-miRNA (Dy547-pre-miR, Thermo Scientific Pierce, Rockford, IL, USA, catalogue ID CP-004500-01-0), were prepared using a double emulsion (w1/o/w2) method adapted from Kelly et al. [45], and is briefly described below. To improve encapsulation efficiency, RNA was condensed with the cationic lipid DOTAP at a nitrogen:phosphate (N:P) ratio of 4:1 using a hydration of freeze-dried matrix method adapted from Wu et al. [46]. 

For a 50 mg PLGA preparation, 8 μg of miRNA was diluted in 200 μL of diethyl pyrocarbonate- (DEPC, Sigma-Aldrich, Dublin, Ireland, D5758)-treated RNase-free water (the equivalent of 24.2 nmoles of phosphate). To improve encapsulation, miRNA was condensed with DOTAP at a N:P ratio of 4:1 using a hydration of freeze-dried matrix (HFDM) method [35,46] (Fifty milligrams of PLGA 503 (Boehringer Ingelheim, Ingelheim, Germany) was dissolved in 1.75 mL dichloromethane (DCM) (2.9% w/v) and vortexed. Lyophilised miRNA/DOTAP was resuspended in 50 μL of RNase-free water and added to 1 mL of the PLGA solution and vortexed vigorously. The remaining 750 μL of PLGA solution was added and the mixture was manually probe-sonicated at 70% frequency setting for 30 s on a Branson SLP Sonifier to form the primary (w/o) emulsion. The primary emulsion was added dropwise to a 5% (w/v) polyvinyl alcohol (PVA) solution (12.5mL) homogenizing over an ice-bath at 13,500 rpm to form a secondary or multiple w/o/w emulsion. This emulsion was added to 25 mL 1% (w/v) PVA and gently stirred for 4 h or overnight at room temperature to allow DCM evaporation. Microparticles were recovered by centrifugation at 1120× *g* for 15 min at 4°C, washed three times with water to remove residual PVA, resuspended in 1.5 mL d H_2_O and then lyophilized (Labconco, FreeZone 4.5, Kansas City, MO, USA).

### 2.2. Microparticle Size Characterisation

Microparticle size was determined by laser diffraction using a Malvern Mastersizer 2000 (Malvern Instruments, Malvern, UK). Lyophilized microparticles (5 mg) were resuspended in 1 mL of HPLC grade H_2_O (Sigma-Aldrich, 270733) and sonicated. Particle size for each batch was acquired three times and means were calculated.

### 2.3. Determination of Zeta Potential of Microparticles

ζ potentials of microparticles were measured in disposable capillary cells using a Zetasizer Nano ZS (Malvern, UK) to determine surface charge. Lyophilized microparticles (1 mg) were resuspended in 1 mL of HPLC grade H_2_O and sonicated. ζ potential for each batch was acquired five times and means were calculated.

### 2.4. miRNA Encapsulation Efficiency

Lyophilised PLGA microparticles encapsulating RNA (1 mg) were dissolved in 200 μL of chloroform. To this, 500 μL of 1 × Tris–EDTA (TE) buffer (10 nM Tris-HCl, 1 mM EDTA, pH 7.5) was added and the mixture was vortexed for 1 h, followed by centrifugation at 15,120× *g* for 20 min at 4 °C. Four hundred microliters of the upper aqueous layer were collected and transferred into another tube. RNA quantification was estimated using a RiboGreen® assay (as described below) or, in the case of fluorescently tagged Dy547-pre-miR (Fl-miRNA), fluorescence intensities were measured using fluorescent spectroscopy at 485_nm_ and 520_nm_ excitation and emission wavelengths, respectively. Fl-miRNA concentration was determined using a prepared calibration curve.

The Quant-iT™ RiboGreen® quantification utilises a fluorescent RNA stain that can provide 200- and 1000-fold greater sensitivity than ethidium bromide and ultraviolet based detection. RiboGreen® reagent binds RNA and fluoresces with maximum excitation and emission wavelengths of 500_nm_ and 525_nm_, respectively. Fluorescence can be detected at fluorescein wavelengths with detection as low as 1 ng/ml RNA [47]. Briefly, 100 μL of appropriately diluted standards and samples in TE buffer were added to wells of black 96-well plates in triplicate. RiboGreen® reagent was diluted 2000-fold in 1 × TE buffer. One hundred microliters of diluted RiboGreen® reagent were added to each well and protected from the light until read at fluorescein excitation and emission settings on a multiplate reader. Fluorescence of blank reagent was subtracted from sample and standard readings as recommended by the manufacturer. Standard curves were prepared using ribosomal RNA (rRNA) standard supplied or RNA of known concentration to give a curve from 1 to 50 ng/mL. Percentage encapsulation efficiency was calculated relative to the initial amount of miRNA added per mg of PLGA by dividing [retrieved miRNA (ng)/mg of PLGA] by [loaded miRNA(ng)/mg of PLGA] × 100. In vitro release and stability studies have previously reported for siRNA PLGA MPs [35].

### 2.5. Differentiation of U937 Monocytes into Macrophages

U937 cells, a human monocytic (histiocytic lymphoma) cell line (ECACC), were cultured in RPMI 1640+GlutaMax (Gibco, Rockford, IL, USA, 61870-010) and differentiated into macrophage-like cells via stimulation with phorbol 12-myristate 13-acetate (PMA; Sigma-Aldrich, P8139), a canonical protein kinase C activator. U937 cells were seeded into 24-well plates at a density of 2 × 10^4^ cells/well and differentiated by the treatment with 50 nM PMA for 48 h, followed by replacement of media and culture for a further 4 days, with feeding after 2 days.

### 2.6. Transfection of PMA-Differentiated U937 Cells with PLGA Microparticles

PMA-differentiated U937 cells were incubated for 3 h with varying doses of PLGA microparticle formulations diluted in OptiMEM I reduced serum media (Gibco) or OptiMEM I alone in a 37°C, 5% CO_2_, humidified environment. Following this incubation, cells were washed with warmed phosphate buffered saline (PBS) and medium was replaced. After various transfection time-points, RNA was isolated using TRI reagent (Sigma-Aldrich, T9424), according to the manufacturer’s instructions, and was resuspended in 0.1% DEPC-treated H_2_O. 

### 2.7. Gene Expression Analysis by Quantitative Real Time-Polymerase Chain Reaction (qRT-PCR)

Quantification of RNA was performed on an 8-channel Nanodrop 8000 spectrophotometer (Thermo Scientific, Rockford, IL, USA). An A_260_/A_280_ ratio of > 1.8 is indicative of pure RNA and was used for subsequent assays. RNA was reverse transcribed into complementary DNA (cDNA) to use as templates for real time (RT)-PCR reactions using the Quantitect® Reverse Transcription Kit (Qiagen, Venlo, The netherlands, 205313). Primers were designed using Primer 3 online software (http://frodo.wi.mit.edu) and Primer-BLAST (http://www.ncbi.nlm.nih.gov/tools/primer-blast) and were obtained from MWG Eurofins Operon (Table 1). 

qRT-PCR was performed on a LightCycler® 480 (Roche, Penzberg, Germany) using a SYBR Green master mix (Roche, 04707516001). The 2^-ΔΔ*C*t^ method was used to quantify the expression of SLPI relative to glyceraldehyde 3-phosphate dehydrogenase (GAPDH) [48]. All qRT-PCR experiments included no-RTase and no-template controls.

### 2.8. TaqMan microRNA Assays

cDNA was generated from extracted RNA for the miRNAs of interest using miRNA-specific stem-loop reverse transcription (RT) primers from TaqMan® MicroRNA assays. RT reactions were performed using the TaqMan® MicroRNA Reverse Transcription kit. Mature miRNA expression was measured with qRT-PCR using TaqMan® MicroRNA Reverse Transcription and has-miR-19b-3p (Assay ID 000396) and U6 snRNA (Assay ID 001973) TaqMan® miRNA assays (Applied Biosystems) according to the manufacturer’s instructions on a LightCycler® 480 mono colour hydrolysis probe programme. 

### 2.9. Statistical Analysis 

All analyses were performed using GraphPad PRISM 4.0 software package (San Diego, CA, USA). Results are expressed as the mean ± SEM and were compared by student *t* test (non-parametric, 1 or 2 tailed) or ANOVA as appropriate. Differences were considered significant at *p* ≤ 0.05. All experiments throughout the study were performed in triplicate unless otherwise stated. 

## 3. Results

### 3.1. Characterisation of PLGA Microparticles

**Figure 1:**
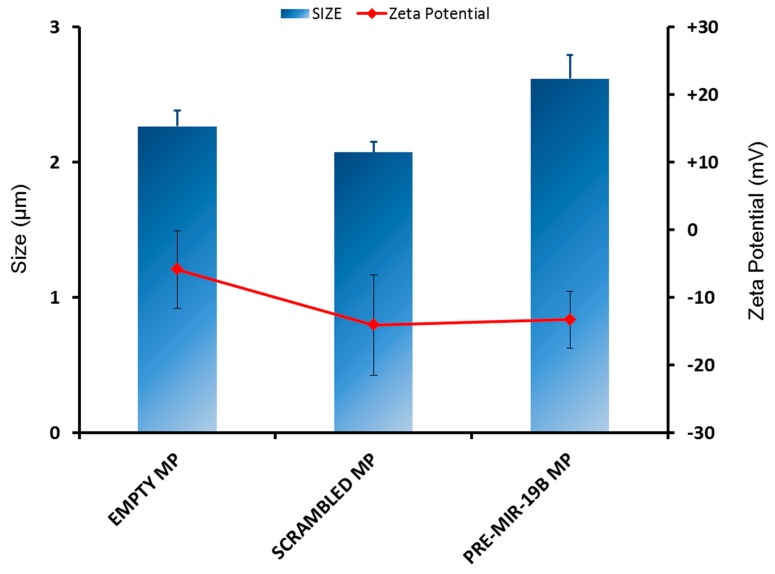
Size and ζ potential of poly (lactic-*co*-glycolic acid) (PLGA) formulations. Empty (no RNA) PLGA microparticles (MP) and scrambled non-targeting- or pre-miR-19b mimic-loaded PLGA MPs were prepared using a double emulsion, solvent evaporation method. Size (volume median diameter, μm) of PLGA MPs was determined in triplicate using laser diffraction (Mastersizer 2000, Malvern, UK), blue bars. ζ potential (mV) of PLGA microparticles was determined (*n* = 5 technical replicates, red line) using Laser Doppler Velocimetry (Zetasizer Nano ZS, Malvern, UK). Data are depicted as mean ± SEM, *n* ≥ 3.

In order to selectively target microRNA mimics to macrophages, pre-miR-19b-3p, scrambled non-targeting pre-miR mimic or Dy547-pre-miR were encapsulated in PLGA-based microparticles using the double emulsion solvent evaporation method. These were then characterised for physicochemical features including particle size, ζ potential and encapsulation efficiency. An important characteristic in the development of a drug delivery system is particle size. Correct size is important to ensure optimal cell targeting and uptake, but additionally aerodynamic diameter of particles is critical to ensure adequate dosing by inhalation [49]. PLGA microparticles of 2–3 μm were previously determined as an optimal size for alveolar macrophage uptake [34]. This size range falls into the inhalable size range of particles, which is generally set at < 5 μm. Size was determined for each microparticle formulation and mean diameters were found to be between 2 μm and 3 μm (Figure 1), which is within the size range for effective pulmonary delivery and macrophage uptake. The span for miRNA-encapsulated PLGA microparticles’ size distribution was 1.02675 ± 0.005123 (SD) μm, indicating the uniformity of the particle sizes. All microparticle formulations had a negative ζ potential between −5 mV and −14 mV (Figure 1). Size distribution and zeta graphs are provided in Supplementary Materials (Figures S1 and S2). 

To accurately assess nucleic acid dosing and concentrations, the amount of miRNA mimics and inhibitors encapsulated into PLGA microparticles was determined. PLGA microparticle encapsulation efficiencies of nucleic acids on average were found to be 26.8 ± 12.9% for all RNA-loaded PLGA-based microparticles (Table 2). No significant differences were observed between the different miRNA mimics in terms of encapsulation efficiency. 

### 3.2. Internalisation and Intracellular Pre-miR-19b-3p Delivery to Macrophages Using Microparticles 

**Figure 2:**
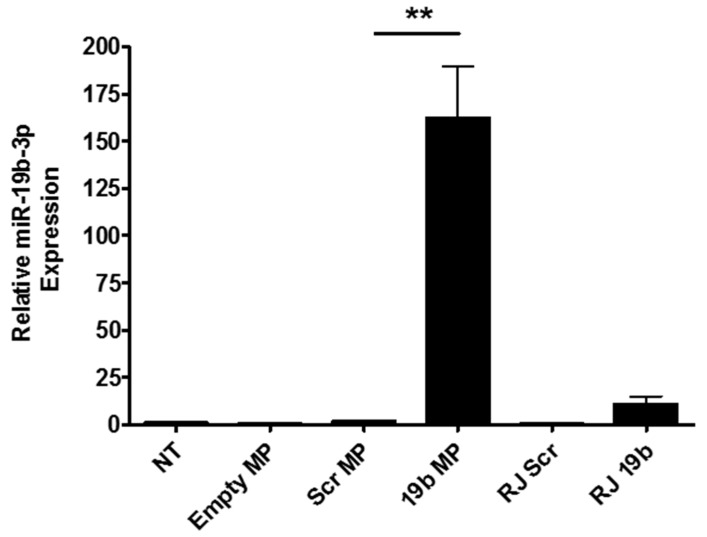
The effects of PLGA microparticles on the levels of mature miR-19-3p in phorbol myristic acetate (PMA)-differentiated U937 macrophages post transfection, as determined by qRT-PCR. Cells were treated, in triplicate, with empty MPs, MPs containing scrambled non-targeting control miRNA (Scr) or pre-miR19b (19b) or RiboJuice™ (RJ) controls at an RNA concentration of 30 nM. Cells were washed and media was replaced after three hours, and cells were lysed at 72 h post transfection. Results (mean + SEM) are normalised to U6 RNA expression. NT: non-transfected, data representative of three experiments (** *p* ≤ 0.01, one-tailed *t* test).

PLGA particles have previously been used by our group to successfully deliver siRNA to macrophages [35]. In that study, a comprehensive screening of their interaction with macrophages was undertaken using high content analysis. Here, we harnessed these PLGA-based microparticles for delivery of a miRNA modulator cargo to macrophages. In order to assess the ability of these microparticles to do this, PMA-differentiated U937 macrophages were transfected with empty PLGA microparticles or those encapsulating pre-miR-19b-3p or scrambled mimics. If internalised and released, these pre-miRs could potentially enter the miRNA pathway and be cleaved by Dicer to form mature microRNA. As a functional measure of transfection efficiency, the levels of mature miR-19b-3p were quantified by qRT-PCR. A dose ranging study indicated that levels of mature miR-19b-3p increased with increasing concentration of microparticles, and 30 nM was selected for subsequent experiments. Another pilot study showed that the highest levels of mature miR-19b-3p were observed 24 h post transfection but were sustained until at least 72 h post transfection. Figure 2 shows that the levels of the mature miR-19b-3p transcript were significantly higher in cells treated with pre-miR-19b-3p-loaded PLGA microparticles compared to empty microparticles, or scrambled mimic controls, indicating successful internalisation and miR-19b-3p release from the microparticle. In addition, the PLGA microparticles were more effective than RiboJuice™. 

### 3.3. Functional Assessment of Pre-miR19b-Loaded PLGA Microparticles on the Expression of SLPI in PMA-Differentiated U937 Macrophages

**Figure 3:**
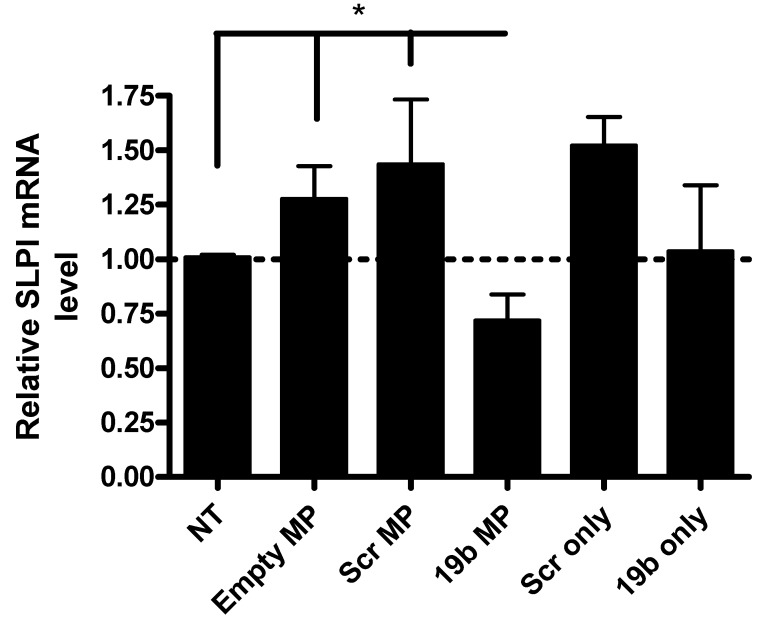
The effects of PLGA microparticle formulations on the expression of SLPI in PMA-differentiated U937 macrophages post transfection, as determined by qRT-PCR. Cells were left untreated (NT) or treated, in triplicate, with empty (no RNA), scrambled control (Scr) or pre-miR19b (19b)-containing PLGA microparticles (MP) at an RNA concentration of 30 nM. Cells were also treated with scrambled control (Scr) or pre-miR19b alone (only). Cells were washed and media was replaced after three hours and cells were lysed 72 h post transfection. Results (mean + SEM) are normalised to GAPDH expression; data are representative of three experiments. NT: non-transfected. *p ≤ 0.05; one-tailed *t* test, compared to 19b MP.

Although pharmaceutical characteristics such as size are important parameters in the development of RNA-based drugs, ultimately it is the functional effectiveness of these formulations that is the key data required at early stage in vitro screening. For this reason, the PLGA microparticles encapsulating pre-miR mimics were transfected into PMA-differentiated U937 macrophages. As efficient internalisation had been observed, target gene expression was then examined to determine the downstream functionality of the sequences in the cells. Preliminary data indicated that the highest target gene (SLPI) knockdown was observed at a later stage (48–72 h) post transfection (data not shown). Significant knockdown of SLPI expression (≥ 29%) was found 72 h post transfection using pre-miR-19b-3p-loaded microparticles compared to empty microparticles and scrambled-containing microparticle PLGA controls (*p* ≤ 0.05) (Figure 3).

## 4. Discussion

Delivery of therapeutics directly to the respiratory tract is a promising modality for non-invasive local treatment of respiratory diseases using microtechnology. It is now well known that aberrant miRNA expression is involved in a range of diseases and in the case of conditions involving an overexpression of miRNA, various strategies are currently being investigated including the use of modified antisense oligonucleotides, specifically antagomiRs [50,51]. Given the fact that miRNAs are key regulators of diverse biological pathways, and that they themselves may be modifier genes for CF and other diseases, their use as therapeutic targets or therapeutics themselves is becoming increasingly examined. For those conditions where under-expression of particular miRNAs is involved, these may be introduced into affected cells as pre-miR mimics. As nucleic acids are large, negatively charged molecules and targeted by many endogenous nucleases, a range of protective vectors have been explored for their delivery, including polymers [49]. Polymeric particles, when engineered appropriately, can facilitate intracellular delivery, enhance in vivo stability and target their delivery [52]. miRNA-based medicines may therefore provide new therapeutic options for the treatment of many disease states, including cystic fibrosis. Here, we explored the usefulness of PLGA for the encapsulation and delivery of miRNA mimics to macrophages.

Microparticles of the optimal size for lung alveolar macrophage delivery, between 2–3 µm in diameter, were prepared using a double emulsion, solvent evaporation method. The ζ potential of all of these microparticles was negative, possibly due to underivatised lactic acid and glycolic acid on their surfaces. Macrophages are excellent phagocytes and are sensitive to ‘foreign’ material, with the ability to produce high amounts of pro-inflammatory cytokines such as IL-8 when stimulated. IL-8 secretion was not stimulated by transfection with PLGA microparticles in this study (data not shown), therefore consolidating the notion that PLGA is useful as a biocompatible polymer.

In vitro, we observed significant uptake of PLGA encapsulated pre-miR mimics by PMA-differentiated U937 macrophages, as determined by quantification of the levels of mature miRs in these cells. This led to a target gene knockdown of almost 30%; however, further optimisation of dosing may be required to increase target knockdown. An issue ultimately affecting this is encapsulation efficiency, which was low. Theoretically, higher encapsulation efficiencies equal higher drug loading, in this case miRNA mimics and inhibitors, and therefore lower PLGA microparticle dosing, thus improving biocompatibility. We achieved an average encapsulation efficiency of approximately 27%. A number of factors may affect encapsulation efficiency. During the preparation of the microparticles, miRNA is subjected to mechanical, thermal and chemical stressors which may lead to damage. Although damage can be caused by sonication, homogenisation, high interfacial surface tension at the oil water interface, and acidic conditions during formulation due to the presence of PLGA, this probably will not affect the encapsulation efficiency a great deal and will more likely affect RNA structure. It is more likely that the RNA is not encapsulated rather than damaged. The stability of the primary (w/o) emulsion is known to be a critical factor for efficient internalisation of the drug in the double emulsion-solvent evaporation method [53,54]. The encapsulation efficiency of an unstable primary emulsion can be low because of the tendency of the internal aqueous phase (w1) to merge with the neighbouring aqueous continuous phase (w2) [55]. The stability of the primary emulsion can be improved by including emulsifying agents such as PVA, BSA or Tween-80, either in the internal aqueous phase (w1) or in the polymer phase (o) [55]. The concentration of PVA used has been directly correlated with encapsulation efficiency of PLGA nanoparticles, which may also be the case for microparticles. This may be related to increasing concentrations of PVA solutions having increased viscosity, leading to the resistance to the outward diffusion of drug from the internal aqueous phase and to the better stabilization of the emulsion [56,57]. Therefore, altering some of these characteristics in the production of miRNA-encapsulating PLGA microparticles may lead to higher encapsulation efficiencies.

Here, we used miR-19b-3p and its predicted target SLPI to demonstrate that encapsulation of a miRNA mimic in a PLGA microparticle can be used for functional delivery to macrophages. In reality, increasing SLPI expression via an antimiR approach rather than inhibiting its expression using a miRNA mimic would be more likely to have therapeutic value for CF, where SLPI’s antiprotease and immune-modulator functions would be of value. Unfortunately, antimiR studies were beyond the scope of the current work. Other work from our group has validated that SLPI is indeed a true target of miR-19b-3p [58]. Furthermore, a recent study of ours using a microRNA capture technique, termed miR-CATCH, also confirms the association between miR-19b-3p and *SLPI* mRNA [59]. This technique allows for the identification of miRNAs targeting the full-length mRNA by ‘pulling-down’ the mRNA of interest along with all cross-linked bound miRNA. Using this technique, miR-19b-3p was found to be bound to native *SLPI* mRNA. 

Local delivery to CF lungs is one of the most promising approaches for bringing miRNA-technologies targeting CF to the clinic. Inhalation offers tissue-specific targeting of the miRNA and minimal systemic exposure, thereby diminishing the risk of off-target effects. CF lungs represent both significant anatomical and pathological barriers to inhaled medicines, however, including obstructed airways covered with thickened mucus and mucus plugs. In order to develop therapeutics such as these for local aerosolised delivery to the CF lung, the next steps will be to evaluate their efficacy in mucus-producing air-liquid interface co-cultures of primary or transformed CF airway epithelial cells and alveolar macrophages. 

In conclusion we developed a PLGA-based microparticle platform for targeted delivery of miRNAs to innate immune cells such as macrophages. Using miR-19b-3p as a proof of principle cargo, this was encapsulated within microparticles of approximately 2 microns in size. These miRNA-based microparticles had the ability to be internalised into monocyte-derived macrophages, with concurrent target gene knockdown. Further studies should focus on increasing the encapsulation efficiency of these microparticles and evaluate cytotoxicity in more depth. Local delivery to the lungs is a promising approach, as it reduces off target effects by enhancing delivery to the site of action. Microparticles are generally prepared as dry powder systems and those outlined in this study were of correct size for deep lung deposition via inhaler devices. With refinement, this platform may become an important tool in the delivery of miRNA-based therapeutics for the treatment of CF and other inflammatory lung diseases.

## Figures and Tables

**Table 1:** Details of primer pairs used in analysis of mRNA expression by qRT-PCR. SLPI: secretory leucoprotease inhibitor.

mRNA	Primers (5’-3’)	Annealing Temperature (°C)	Product Size (bp)
GAPDH	(F)- CATGAGAAGTATGACAACAGCCT (R)- AGTCCTTCCACGATACCAAAGT	57	113
SLPI	(F)- AATGCCTGGATCCTGTTGAC (R)- AAAGGACCTGGACCACACAG	57	243

**Table 2:** Summary of percent encapsulation efficiency of PLGA microparticles (means ± SD).

Microparticle Formulation	Encapsulation Efficiency (%)
Unloaded MP	N/A
pre-miR-19b-MP	37.6 ± 13.37
scrambled mimic-MP	24.53 ± 9.65
Dy547-pre-miR-MP	23.2 ± 8.96

## Supplementary Materials

The following are available online at http://www.mdpi.com/2305-6320/5/4/133/s1, Figure S1: Representative size distribution of PM19b MP, Figure S2: Representative zeta graph of PM19b MP.
