# Supplementary material for: Knockdown of Gene Expression in Macrophages by microRNA Mimic-Containing Poly (Lactic-co-glycolic Acid) Microparticles
Source: Medicines (Basel). 2018 Dec 15;5(4):133. doi: 10.3390/medicines5040133 (PMC6313440; doi:10.3390/medicines5040133)
Supplement: Supplementary file 1 [file medicines-05-00133-s001.pdf]

Supplementary Materials: Knockdown of Gene Expression in Macrophages by microRNA Mimic-Containing Poly (Lactic-*co*-glycolic Acid) Microparticles

Paul J. McKiernan, Patrick Lynch, Joanne M. Ramsey, Sally Ann Cryan and Catherine M. Greene

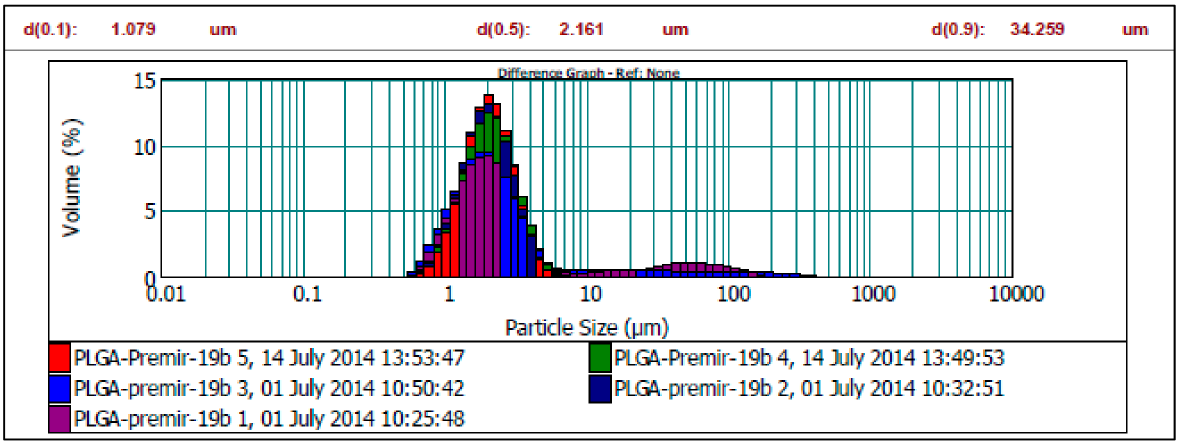

Figure S1. Representative size distribution of PM19b MP.

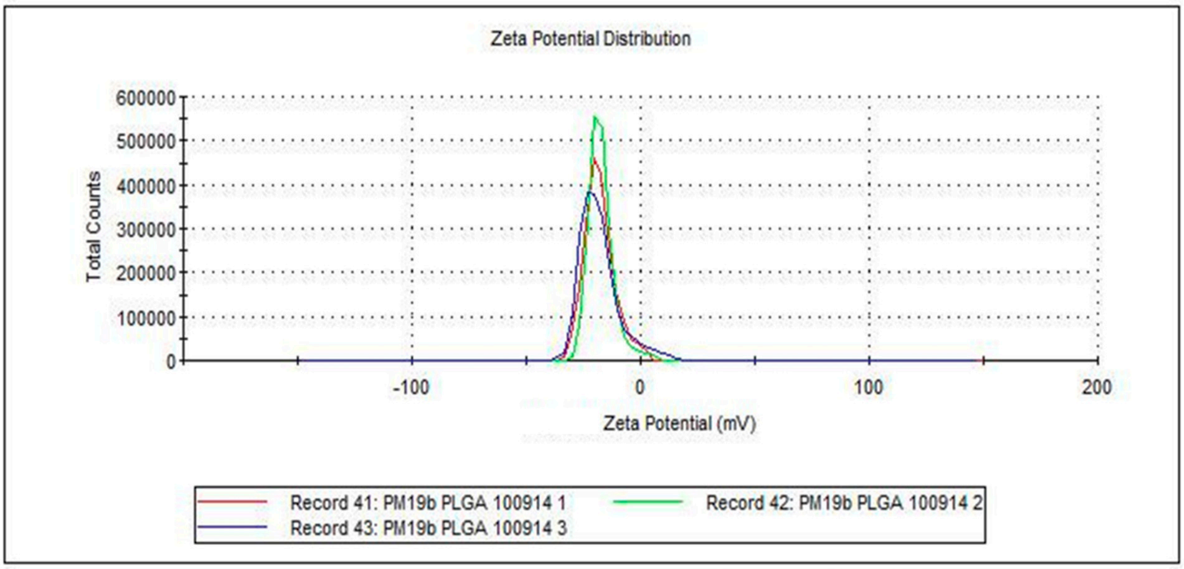

Figure S2. Representative zeta graph of PM19b MP.
